# Thermo-Responsive Hydrogels: From Recent Progress to Biomedical Applications

**DOI:** 10.3390/gels7030077

**Published:** 2021-06-24

**Authors:** Kaiwen Zhang, Kun Xue, Xian Jun Loh

**Affiliations:** 1 Department of Biomedical Engineering, College of Engineering, Southern University of Science and Technology (SUSTech), 1088 Xueyuan Avenue, Shenzhen 518055, China; 11712821@mail.sustech.edu.cn; 2 Institute of Materials Research and Engineering (IMRE), Agency for Science, Technology and Research (A*STAR), 2 Fusionopolis Way, Singapore 138634, Singapore; 3 Department of Materials Science and Engineering, National University of Singapore (NUS), 9 Engineering Drive 1, Singapore 117576, Singapore

**Keywords:** in situ hydrogel, thermogel, supramolecular hydrogel, LCST polymer, biomedical

## Abstract

Thermogels are also known as thermo-sensitive or thermo-responsive hydrogels and can undergo a sol–gel transition as the temperature increases. This thermogelling behavior is the result of combined action from multiscale thermo-responsive mechanisms. From micro to macro, these mechanisms can be attributed to LCST behavior, micellization, and micelle aggregation of thermogelling polymers. Due to its facile phase conversion properties, thermogels are injectable yet can form an in situ gel in the human body. Thermogels act as a useful platform biomaterial that operates at physiological body temperatures. The purpose of this review is to summarize the recent progress in thermogel research, including investigations on the thermogel gelation mechanism and its applications in drug delivery, 3D cell culture, and tissue engineering. The review also discusses emerging directions in the study of thermogels.

## 1. Introduction

Hydrogels are generally a series of relatively hydrophilic polymers with the ability to form a three-dimensional crosslinked network and preserve a large amount of water. Due to their high water content, hydrogels show similar properties with human body tissue and higher biocompatibility, which makes them suitable for biomedical applications [1,2,3]. Based on the type of crosslinked bond, hydrogels can be classified into chemically crosslinked hydrogels and physically crosslinked hydrogels. Chemically crosslinked hydrogels form a permanent crosslink which is non-reversible. Within a physically crosslinked hydrogel, supramolecular hydrogels are crosslinked via noncovalent bonds such as host–guest interactions, ionic interactions, ligand coordination, and hydrogen bonding interactions [4,5]. Due to dynamic non-covalent interactions, supramolecular hydrogels show features such as reversibility, repairability, responsiveness and other desirable properties [6,7]. This results in the possibility to design ‘smart’ hydrogels which can sense environmental changes and respond to the change, such as through pH response, photo-response, and temperature response. In the history of biomedical materials, those of the first generation exhibit biological inertness, while the second and third generation materials are bioactive and can stimulate cellular response [8]. Supramolecular hydrogel are promising materials known as the fourth generation of biomedical materials with the ability to mimic the extracellular matrix and are smart materials which can respond to extracellular stimuli [9]. The great potential of supramolecular hydrogels has attracted tremendous interest from researchers and they have been used for numerous biomedical applications in the past decade.

Thermogels, or thermo-responsive hydrogels, are a subclass of the supramolecular hydrogels that gelate via hydrophobic interactions. Thermogels can undergo a sol–gel phase transition because they are constituted of amphiphilic polymers with both hydrophilic parts and hydrophobic parts [10]. Thermogels typically refer to thermosensitive hydrogels which can form a gel at a higher temperature and return back to a liquid at a lower temperature within a certain temperature range, which is contrary to the conventional melt transition behavior [11]. This gelation procedure does not need any other assistance or other triggers such as enzymes; thus, it is considered as a benign phase conversion procedure. The lack of toxic crosslinking agents renders it more likely that thermogels would show intrinsic biocompatibility as an injectable in situ hydrogel [12,13]. Considering thermogel polymers used in biomedical application, two main categories can be identified on the basis of biodegradability: Firstly, non-biodegradable thermogels, which include (1) polyacrylates and (2) Pluronic^®^. Secondly, biodegradable thermogels, which include (1) polyesters, (2) polypeptides, and (3) polysaccharides [11,14,15,16]. To undergo phase transition from room temperature to body temperature, thermogels that can undergo gelation within the range of 25–37 °C are especially valuable in the biomedical field. Due to phase transition at physiological temperatures, thermogels have been used in diverse biomedical applications [17,18].

In this review, we summarize the recent progress of thermogel research. In particular, we review investigations pertaining to thermogel gelation properties and the innovative use of thermogelling systems for biomedical applications including drug delivery, 3D cell culture, and tissue engineering. Finally, we provide a perspective on the future development of thermogels.

## 2. Thermogelling Mechanism/Thermogelling Properties

The gelation mechanism of thermogels, or thermoresponsive hydrogels, depends on many factors that affect its properties. In this section, we introduce some important concepts in thermogelling systems and briefly review the recent progress focused on thermogelling mechanism from the most fundamental polymer level all the way to the more macro hydrogel level.

### 2.1. LCST

**Figure 1:**
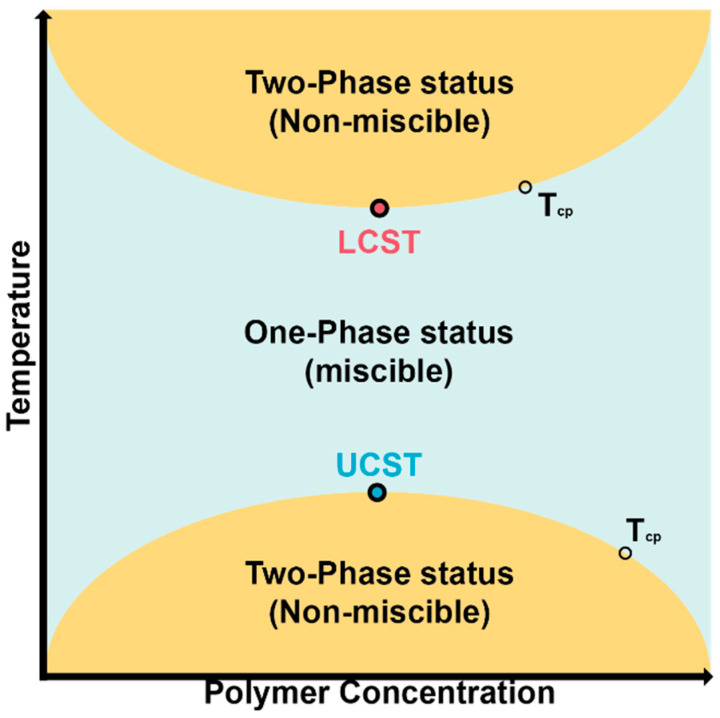
LCST and UCST of thermosensitive polymers.

Lower critical solution temperature (LCST) and upper critical solution temperature (UCST) behavior are important terms when describing thermoresponsive polymers; they are also the underlying instructive concept for the design of thermogels. For polymers with UCST, they are miscible in water above the UCST and immiscible below. Though broadly speaking, polymers with UCST behavior can also be defined as thermogelling polymers, there are fewer studies which reported their application as thermogels in the biomedical field because they are only injectable at a higher temperature that may denature some loaded drugs especially some polypeptides and proteins [19]. Some supramolecular rotaxane hydrogels form a UCST type sol-to-gel transition and have been used in the controlled release of drugs and constructing self-healing hydrogels, and we direct interested readers to these systems [20,21]. UCST type thermogels will not be discussed further in this review. In contrast, if a polymer shows a change from miscible to immiscible in the water while heating, then LCST describes the minimum temperature required for the polymer to form into a gel in the system [22]. As Figure 1 shows, the phase transition temperature of LCST polymers changes with polymer concentration. Only the lowest temperature can be called LCST, while other temperatures above LCST but which also induce phase change are called the cloud point temperature (T_cp_), which is sometimes misused as LCST. Hoogenboom and colleagues clarified the definition and recommended an optimized measurement condition of T_cp_ for LCST polymers [23]. As there are increasing number of studies involving LCST, standardizing the characterization condition is important for further study of LSCT polymers. To date, numerous polymers with LCST behavior have been found [24], and Table 1 lists the LCST of some typical and commonly used thermosensitive polymers. 

Among the polymers listed above, poly(N-isopropylacrylamide) (PNIPAM) is the most frequently studied thermogelling polymer with a LCST. PNIPAM is a homopolymer that has a low cytotoxicity and a LCST around 32 °C, which makes it suitable for physiological thermoresponsiveness, and the phase separation behavior has been extensively investigated. Researchers know that PNIPAM undergoes a coil-to-globule transition via shrinkage of polymer chain due to the hydrophobic effect and a net increase in entropy of the system [33]. Even though the polymer undergoes a sol-to-gel transition from disorder to order, the surrounding water molecules are able to move freely with the coil-to-globule transition, thus increasing the overall entropy of the system. Recently, several studies have revealed the interaction mechanism involving PNIPAM itself and PNIPAM/water. Kinoshita and colleagues used a statistical mechanical theory to investigate the energetics and entropy changes in the reverse globule-to-coil transition, and found that the mechanism is similar to the cold denaturation of proteins [34]. Paulo et al. found that the collapse of PNIPAM chain at high temperatures is related to the reduced coordination of both amide group and isopropyl group with water, and is observed together with an increase in the monomer–monomer hydrogen bond [35]. The thermodynamics of NIPAM monomer was investigated by Heyda et al. [36], which could allow for better control of PNIPAM phase behavior. Nellas and coworkers performed a molecular dynamics simulation of pentamer PNIPAM [37], and they suggest a clathrate-like behavior in the coordinate shell might be responsible for the LCST behavior of PNIPAM. Tang et al. directly observed the hydrophilicity–hydrophobicity transformation of PNIPAM by the utilization of aggregation-induced emission luminogens [38]. In that study, it was found that the hydrophobicity change of PNIPAM chains was due to the formation of multiple interchain/intrachain hydrogen bonds. As we can see, multiple advanced technologies have been used to investigate the mechanism of LCST polymers. These studies give us a better understanding of thermogelling homopolymers and inspire the design of composite thermoresponsive hydrogels. Furthermore, the LCST value of PNIPAM can be tuned by copolymerizing with other hydrophilic/hydrophobic monomers together with PNIPAM. Adding more hydrophilic monomers such as hydroxyl ethylacrylamide (HEAm) could make the LCST increase to 50 °C [39], while hydrophobic monomers such as tertbutyl acrylamide (TBAM), N-tert-butylacrylamide (NT), or butylacrylate (BA) could lower the LCST and lead to a stiffer gel [40,41]. Nonuniform shrinkage caused by a specific spatial arrangement of PNIPAM-based polymers with different LCST can lead to macroscopic bending, folding, twisting, and other shape transformations of the hydrogel at different temperatures, with potential use as a smart soft actuator [42]. However, PNIPAM is non-biodegradable and there are still some conflicting viewpoints about its biocompatibility [15,43]. Recent studies in the biomedical field are mainly focused on PNIPAM copolymer or composites, which can partly solve the degradation problem of PNIPAM, such as chitosan-grafted-PNIPAM [44,45] and peptide-grafted-PNIPAM [46]. PNIPAM is still a promising material for conferring thermoresponsiveness to a composite hydrogel. Compared with homopolymers, due to its complexity and diversity, there are few in-depth reports about LCST mechanism of block copolymers.

### 2.2. Polymer Configurations and Micelle Properties

**Figure 2:**
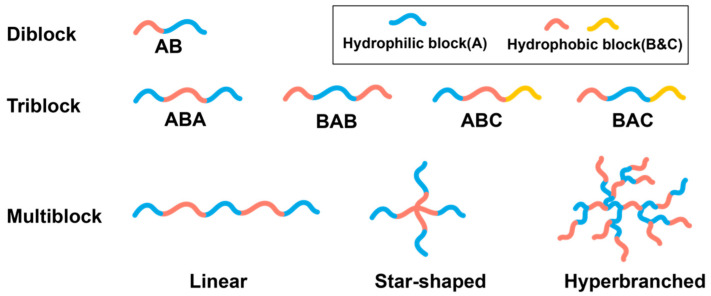
Polymer configurations of thermogel copolymers.

Except for PNIPAM and some chitosan-based thermogels, thermogelling polymers are generally amphiphilic block copolymers. Thermogelling amphiphilic block copolymers can have varied configurations and may be classified according to block number into diblock, triblock, and multiblock. For triblock copolymers, they can be further categorized into ABA type, BAB type, ABC type, and BAC type, where ‘A’ represents the hydrophilic block, and ‘B’ and ‘C’ represent the hydrophobic block. Triblock amphiphilic copolymers are predominant in thermogelling systems nowadays, and the two most represented are the Pluronic family (e.g., Pluronic F-127 [14]) and PLGA/PEG family (E.g. PLGA-PEG-PLGA [47]). Additionally, multiblock copolymers can also be divided into linear [48], star-shaped [49], and hyperbranched [50,51] (Figure 2). Star-shaped and hyperbranched thermogels are less mentioned in literature. Early studies indicate these two configurations may significantly reduce the gelation concentration and enhance the mechanical strength of thermogel [52]. In contrast, recent research demonstrates that branched polyurethane thermogels may result in an increase of CMC value due to branches sterically blocking the necessary polymer chain interactions to form micelles [53]. More research still needs to be conducted on hyperbranched copolymers as a thermogelling material.

Micellization is the unique behavior of amphiphilic molecules in aqueous solution. This behavior of self-assembly is thermodynamically favorable as long as the thermogelling polymer solution achieves a critical concentration and a critical temperature. Critical micelle concentration (CMC) and critical micelle temperature (CMT) describe the minimal concentration and temperature when amphiphilic molecules convert from unimers to micelles in water. In thermogelling polymer self-assembly behavior, insertion-expulsion and fusion-fragmentation have pivotal roles in micelle equilibrium dynamics. Before reaching equilibrium, copolymer molecules undergo a fast process where the free unimers quickly insert into existing micelles. Subsequently, slower insertion-expulsion and fusion-fragmentation processes dominate micellization [54]. Molecular weight and polymer configuration could also influence the micelle formation. Using Pluronic as an example, short-chain Pluronics could further grow to worm-like micelles, while long-chain Pluronics tend to form spherical micelles [55]. A subfamily of Pluronics, called Tetronic, has a star-shaped configuration and a central diamine group. These polymers have similar micellization behavior as linear Pluronics at normal pH, but micelles become smaller and only form at higher temperatures at acidic pH, while gelation is fully suppressed at highly acidic pH [56]. After micelles are formed, the stability of the micelles may be affected by many factors. It is generally believed that the stability of micelles is directly related to the hydrophobicity of copolymers, and copolymers with strong hydrophobicity tend to have lower CMC. The more hydrophobic the blocks, the stronger the hydrophobicity and the denser the accumulation of hydrophobic cores, leading to the higher stability of micelles. At the same time, the ratio of hydrophilic and hydrophobic segments is also important, if the hydrophilic corona cannot completely wrap the hydrophobic core, the exposure of the hydrophobic core tends to destabilize the micelles [57]. Chain exchange behavior also exists between micelles. In a chain exchange research of triblock copolymer micelles, isotope label and time-resolved small-angle neutron scattering (TR-SANS) were utilized to investigate chain exchange rate [58]. Their result suggests the chain exchange in poly(ethylene-alt-propylene) (PEP) and poly(styrene) (PS)-based PEP-PS-PEP (ABA type) copolymer is much quicker than PS-PEP-PS (BAB type) copolymer in dilute micelle solutions. Another research work suggests that the chain exchange rate will slow down as the molecular weight of the hydrophilic part increases [59]. These studies demonstrate that chain length, block length, and copolymer configurations will affect micelle kinetics and thermodynamics. Furthermore, a change in micelle formation could also influence the gelling mechanism. Since micellization is a fundamental self-assembly behavior of thermogels, in the next part, we will discuss the secondary self-assembly mechanisms that trigger gelation.

### 2.3. Gelling Mechanism

**Figure 3:**
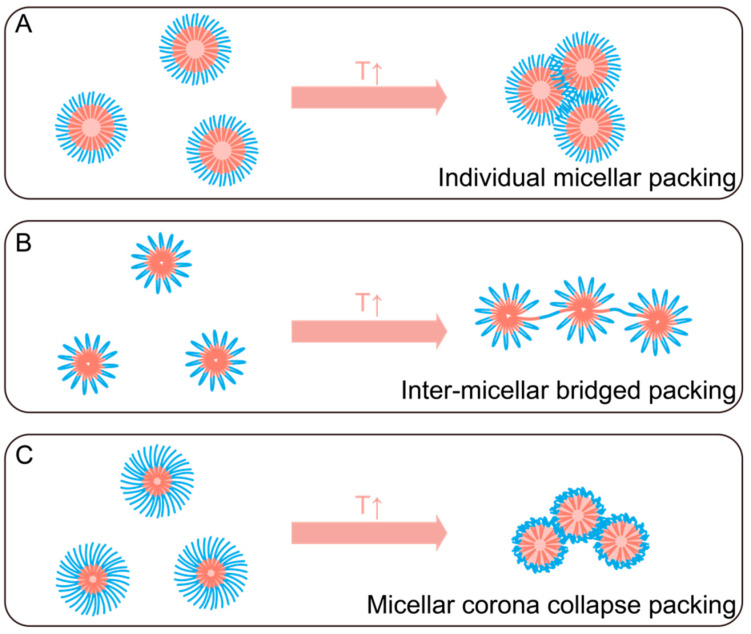
Micelle aggregation type of different thermogel copolymer: (**A**) individual micellar packing, (**B**) inter-micellar bridged packing, and (**C**) micellar corona collapse packing.

The final gelling mechanism of thermogel polymer is attributed to micelle aggregation, which is a more macroscopic thermoresponsive self-assembly behavior. Similar to CMC and CMT above, which relates to micellization, the minimum concentration and temperature required for conversion from micelles to hydrogels are denoted by the critical gelation concentration (CGC) and critical gelation temperature (CGT). When both conditions are reached, micelles begin to pack together tightly to form a three-dimensional network. The porous network structure entraps water inside, and a sol-to-gel transition can be observed. A slight difference of packing mechanism exists between different types of thermogel polymer micelles: (1) individual micellar packing, (2) inter-micellar bridged packing, and (3) micellar corona collapse packing (Figure 3). Individual micellar packing happens on low molecular weight diblock and ABA triblock copolymers. When temperature increases, the hydrophobic force simply drives micelles to aggregate with each other. Short-chain Pluronic follows this aggregation, where the entanglement of the hydrophilic part forms the physical crosslink [60]. Inter-micellar bridged packing may happen for BAB, BAC triblock, and multiblock copolymers. In this case, hydrophobic segments of the polymer chains are connected to two or more micelles, which could lead to a lower CGC and result in a stronger association in thermogelation [61]. Micellar corona collapse packing could happen on copolymer which use polymer with relatively lower LCST such as PNIPAM. The micelles with collapsed corona tend to collide into each other, resulting in quick aggregation [62]. For example, PNIPAM/PHB block copolymer showed outer PNIPAM corona collapse-induced hydrophobicity increase which leads to micelle aggregation and gelation [63]. 

**Figure 4:**
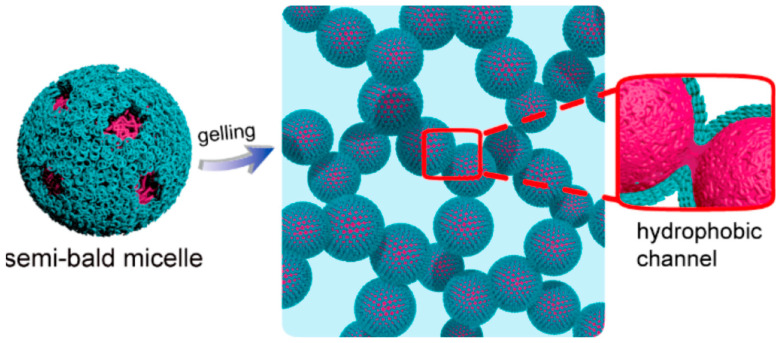
Semi-bald model of PEG/PLGA thermogel. Copyright © 2018, American Chemical Society [66].

Although amphiphilic copolymer micellization is common, amphiphilic copolymer thermogelation is rarer. The gelling mechanism is strictly restricted by factors such as polymer molecular weight and segment composition. For the copolymer with long hydrophilic parts, the micelle shows no thermogelling behavior. The loose corona blocks micelle aggregation and only forms a viscous solution [64]. Fluorescence resonance energy transfer (FRET) imaging is a fluorescence phenomenon that detects intermolecular distances in the nanoscale. Liu et al. used this technique to track the assembly and shedding behavior of micelles from thermogels in vitro and in vivo and suggested that it is feasible to track micelles at the molecular level using FRET [65]. The crosslinking points between thermogel micelles are previously unidentified, while recent research from Ding’s group presented a new mechanism for poly(ethylene glycol)/poly(lactide-co-glycolide) (PEG/PLGA) diblock copolymers during micelle aggregation via FRET and computer simulation method [66]. They suggest that the outer PEG corona of copolymer micelle partially shrinks and folds together to form a semi-bald model where the hydrophobic core is completely exposed in the bald area. We do recognize that the third type of micellar packing by corona collapse shown in Figure 3 also involves the exposing of hydrophobic regions and has similarities to the semi-bald model. As the crosslinking point, the bald zone tightly connects adjacent micelles and forms the hydrophobic channel, which also explained why thermogels could preserve their shape in a large amount of water (Figure 4). The authors performed additional work which reveals the mechanism of PEG/PLGA ABA and BAB triblock thermogel using a similar research methodology [67]. The bridge packing of BAB PEG/PLGA thermogel endows two gel phases with a clear boundary. The semi-bald and bridge crosslinks form the primary gel phase, then as temperature increases the hydrophilic bridges shrink, and the hydrophobic channel becomes the major crosslink point to form secondary stronger gel phase.

## 3. Biomedical Applications

The phase transition from sol-to-gel inspired numerous applications of thermogels, or thermoresponsive hydrogels, in the biomedical field. The range of drugs that could be delivered via thermogels is wide: it has been shown that the loading and release of proteins, DNA, and antibiotics are all compatible with thermogels [68,69,70]. In addition to injectability and easy drug loading in sol phase, there are additional benefits of localized and sustained release. While drug delivery is a common usage of an in situ depot, other applications have also utilized the in situ depot forming capability of thermogels. The 3D crosslinked network of thermogels with localized chemical moieties can satisfy the requirements for 3D cell culture, and bioactive interactions coupled with suitable mechanical properties can render thermogels as an effective tissue engineering scaffold. In this section, recent advances of thermogel in the aforementioned applications will be reviewed. 

### 3.1. Drug Delivery

As more drugs are developed and discovered, cures of previously untreatable diseases become possible. In addition to the drug itself, how the drug is delivered is also key to therapeutic efficacy. Primary methods for drug delivery include oral and direct injection. To reach the therapeutic effect, especially for local disease, maintaining the drug concentration in the therapeutic window is essential. However, drug overdoses could happen. In addition, the short half-life of some drugs limits their efficiency and leads to repeated doses and issues with patient compliance. Table 2 shows a summary of thermogel systems used for drug delivery. Eye drops are the primary method of non-invasive drug delivery to the eye. However, the contact time of common eye drops with the eye is short, and the drug delivery efficiency is low. Hanes and Ensign et al. extended ocular drug delivery time via adding Pluronic F-127 into eye drops [71]. It forms a thin, uniform, clear gel on the ocular surface that could slowly release hydrophilic, hydrophobic, and peptide drugs into the eye. The thermosensitive eye drops could avoid repeated doses, thereby reducing eye irritation and increasing patient compliance. Type 2 diabetes is a common disease that could lead to cardiovascular and cerebrovascular dysfunction and even death [72]. Limited by cost and short half-life, the current peptide drugs show poor performance in chronic clinical treatment. In contrast, liraglutide (an antidiabetic polypeptide) loaded into poly(ε-caprolactone-co-glycolic acid)-poly(ethylene glycol)-poly(ε-caprolactone-co-glycolic acid) (PCGA-PEG-PCGA) thermogels prolong the effective time of one subcutaneous injection [73]. In vivo testing shows the sustained release of liraglutide, which ensures the glucose tolerance of mice was enhanced for a week. The highly flexible nature of PCGA-PEG-PCGA copolymer enhanced mobility of liraglutide in the micelle which increased release yield compared with PLGA-PEG-PLGA thermogels. Ha et al. developed a human C-peptide-loaded elastin-like biopolymer-conjugated thermosensitive hydrogel to treat diabetic complications [74]. The injectable thermogel depot system implements long-time prevention of diabetes-induced inflammation and apoptosis.

Protein and other bioactive factor drugs are considered to be environmentally sensitive and easily denatured, so the delivery of those drugs needs to be precisely controlled with the bioactivity retained. Lee et al. designed a thermal/pH dual sensitive poly (ethylene glycol)-poly (sulfamethazine carbonate urethane) (PEG- PSMCU) copolymer for lysozyme delivery [75]. The PEG-PSMCU hydrogel system shows very low cytotoxicity even in a high copolymer concentration, and further in vivo trials demonstrated that thermogel depot was successfully formed to release protein in rats. Vascular endothelial growth factor (VEGF) is critical when inducing angiogenesis. A nanodiamond (ND)-based composite gelatin chitosan thermogel was reported to provide the sustained release of VEGF and prolonged its activity [76], which offers a potential strategy for growth factor therapy. VEGF proliferation may also lead to dysfunction of normal immune reaction. In a study of a poly(ε-caprolactone)-based polyurethane thermogel, anti-VEGF antibodies were loaded and delivered in the eye to treat proliferative vascular diseases in a rabbit model [77]. The delivery rate and initial burst release could be controlled by adjusting the hydrophilic/lipophilic balance, and a linear continuous release was observed within 40 days after injection. Ding et al. applied the PLGA-PEG-PLGA thermogel to transdermal 5-aminole-vulinic acid(ALA) delivery [78]. The system automatically constructs an asymmetrical structure which converts the skin-touching surface into sol to quickly deliver the drug, and gelation in air to maintain shape, which leverages the advantages of both the sol–gel and gel–sol phase transition properties.

**Figure 5:**
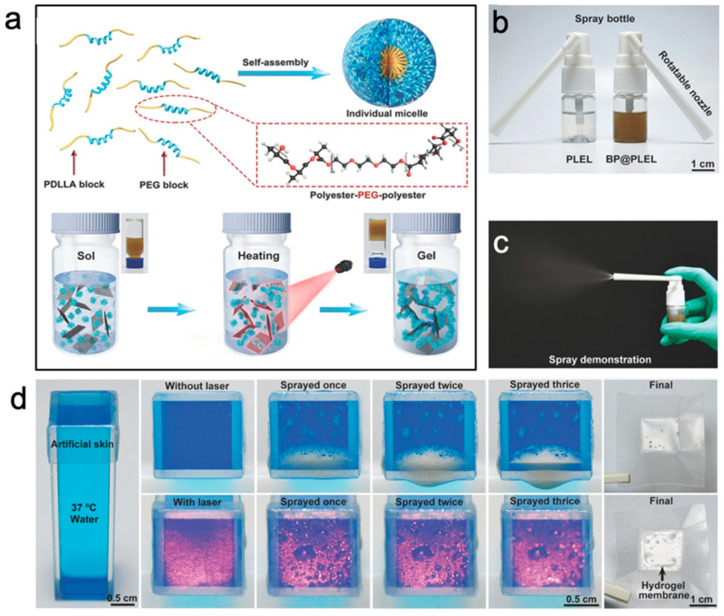
(**a**) Schematic diagrams of BP-loaded PDLLA-PEG-PDLLA thermogel and gelation upon irradiation with near-IR light. (**b**,**c**) Spray demonstration of PDLLA-PEG-PDLLA thermogel. (**d**) Sprayed gel rapidly forms gel layer on artificial skin after NIR irradiation, shown in bottom panel. Taken from [84].

Anticancer drugs such as doxorubicin (DOX), paclitaxel (PTX), etc., have been developed but are limited by their systematic toxicity or poor solubility [79,80]. DOX-loaded alginate-g-PNIPAM thermogel encapsulates the drug within the micelle, and the release profile showed enhanced cellular uptake and overcoming of cancer cell drug resistance [81]. A poly[(R)-3-hydroxybutyrate] (PHB)-based biodegradable polyester thermogel was designed [82], where both DOX and PTX were easily loaded and released locally to treat hepatocellular carcinoma. In vivo tests indicate that this delivery system effectively inhibits and shrinks tumor size. These gel depots can help to treat while minimizing side effects for the tumors when surgery is insufficient. Photothermal therapy is also an effective means of treating tumors [83]. Yu and coworkers designed a poly(d,l-lactide)-poly(ethylene glycol)-poly(d,l-lactide) (PDLLA-PEG-PDLLA)-based thermogel incorporated with black phosphorus (BP) (Figure 5) [84]. Sprayable sol phase thermogel undergoes a quick phase transition to form a solid phase gel film on the wound after tumor removal surgery and shows excellent photothermal performance under NIR light to remove any residual cancer cells. Anticancer therapy can be mediated by multiple different forms of thermogels and plenty of remarkable progress have been made in the field. However, a human clinical trial using an approved thermogel drug delivery system named “OncoGel^TM^”(BTG International Inc, Conshohocken, PA, United States) to treat esophageal cancer was terminated due to the lack of significant improvement and unsatisfactory patient compliance [12,85]. This indicates that even in a well-studied experimental drug delivery system, there is still a long way to go from laboratory to clinic. 

### 3.2. Three-Dimensional Cell/Stem Cell Culture

Traditional cell culture is based on the growth of cells on a 2D platform, which generally cannot represent the real status of cells in their original body tissue. The 3D crosslinked network constructs a porous structure in thermogels which endows it with scaffold-like property and ECM-like nature. In the early years, chondrocytes were reported to be cultured in 3D formed thermogels [86]. Compared with the spindle-like morphology in 2D culture, the chondrocytes in the 3D thermogel culture system expressed more type 2 collagen and tended to form a spherical phenotype, which is important to avoid forming fibrochondrocytes and for maintaining their cartilage regeneration function [87]. A similar situation occurs in stem cell culture, where culturing stem cells on a 2D platform or stiff scaffold could lead to the permanent loss of their cellular differentiation ability [88]. Jeong and colleagues have been working on thermogel 3D stem cell culture. A PEG-PPG-PEG triblock copolymer connected with pyridine-dicarboxylate (PDC) was utilized to culture tonsil-tissue-derived mesenchymal stem cells (TMSCs) by the group [89]. PDC in the gel acts as a ligand to coordinate with metal ions, tune gel modulus, and allow cells to form a suspended spheroid. In testing biomarkers of TMSCs, cell expression demonstrated more stemness in the thermogel system as compared to a 2D system. In the last 5 years, they also studied polypeptide thermogel poly (ethylene glycol)-poly (L-alanine) (PEG-PA) as a base material for TMSCs, with additions such as hepatogenic differentiating factors [90], 2D nanomaterials such as hexagonal layered double hydroxides (LDHs) [91] and graphene and graphene oxide [92] in the system to control differentiation. This is an area that has not been explored sufficiently, and there is potential for more types of thermogels to be tested in this application.

### 3.3. Tissue Engineering

Developing smart scaffolds with the ability to guide tissue regeneration is the key aspect in tissue engineering. Hydrogels with advanced properties have the ability to mimic the structure and biological properties of the native ECM. These scaffolds provide mechanical, spatial, and biological signals to regulate and direct cell adhesion, migration, differentiation, and proliferation for tissue regeneration [93]. The injectability of thermogel systems is an important advantage in designing scaffolds, which confers properties that can conform to any shape, easily load stem cells or growth factors, and have adjustable viscoelasticity. Thermogels as scaffolds have been used in the treatment of many different tissues/organs, and a summary of thermogel systems for tissue engineering is shown in Table 3. Aghdami and Baharvand et al. investigated the feasibility of an electro-conductive gold nanoparticle (GNP)-loaded chitosan thermogel for cardiac repair [94]. Conductive properties can be tuned by the concentration of GNPs and could induce stem cells to differentiate towards adjacent cardiac cells by increasing their electrical coupling. In another study, a PNIPAM thermogel containing PLGA-encapsulated PVP/H_2_O_2_ microspheres was utilized to repair cardiac cells after myocardial infarction (MI) [95]. After 4 weeks of injection in MI area, cardiac cells had reduced expression of transforming growth factor beta (TGFβ) and TGFβRII primers and cardiac fibrosis was attenuated, which indicates that myocardial cells were repaired due to oxygen uptake.

Nerve regeneration is relatively complicated and remains a challenge in tissue engineering. The low regeneration rate and the irreversible nature of neuronal fracture limits the efficiency of treatment for nerve injury [93]. The use of thermogel to induce differentiation of stem cells into neuronal cells has been highly effective in treating nerve injury. Heparin-poloxamer (HP) thermosensitive hydrogels were recently applied to nerve regeneration. HP thermogel achieves peripheral nerve regeneration in the treatment of crushed sciatic nerve injury in a diabetic rat model [96]. Basic fibroblast growth factor (bFGF) and nerve growth factor (NGF) were loaded in the thermogel and released to induce the regeneration of neurilemmal cells. This successfully recovered partial motor functions of the rat. Another work reported the application of HP thermogel in spinal cord injury (SCI) therapy [97], where glial cell-derived neurotrophic factor (GDNF) binding with HP endows the thermogelling system with neuroprotection function to inhibit apoptosis and proliferate neural stem cells.

Bone repair by stem cell-loaded thermogel scaffold is now one of the most promising strategies. The main goal of injectable hydrogels for cartilage and bone tissue engineering is to design bioactive scaffolds with high biocompatibility, biodegradability, stability, and favorable three-dimensional cell culture [98]. Because thermogels are sensitive to temperature stimuli, they can be easily injected and formed in situ, and the mild gel transition conditions confer high biocompatibility, allowing them to be loaded with chondrocytes and growth factors. A poly(N-isopropylacrylamide-co-acrylic acid) (P(NIPAAM-AA)) system was employed as the carrier of rabbit bone mesenchymal stem cells (BMSCs) for osteochondral regeneration [99]. The thermogel system enhanced chondrogenesis-related gene expression in BMSCs, and show a smooth, seamless cartilage repair result under micro-CT scanning for in vivo test in rabbits. It was also reported that the PLGA-PEG-PLGA system could repair full-thickness cartilage by loading BMSCs [100]. Biomechanical test results suggest the newly regenerated cartilage by thermogel-BMSCs system reached 80% of expected Young’s modulus, which is significantly better than the control group with only gel scaffold. In another study, Yu et al. used thermosensitive chitosan hydrogel (CSG)-encapsulated BMSCs and combined with 3D printed poly(caprolactone) (PCL) scaffolds for bone tissue engineering [101]. Despite the many advantages of physically injectable hydrogels, the disadvantages of lower mechanical strength and stability still limit their further application. The addition of 3D printed PCL enhances the mechanical strength of the hybrid scaffold system, where greater cell regeneration was demonstrated by growth factor release and observed by confocal microscopy. Meanwhile, as a 3D print ink, thermogel itself also was reported to be directly used as a 3D print scaffold [102,103]. As 3D printing technology matures, it becomes possible to precisely tune scaffold structures at the nano- and micro-scale. Mimicking tissue anatomy can push thermogel application a step forward in terms of in situ tissue engineering and the construction of in vitro tissue models. However, thermogel with both good printability and biocompatibility are still not yet developed, and new 3D printable thermogels are needed.

**Figure 6:**
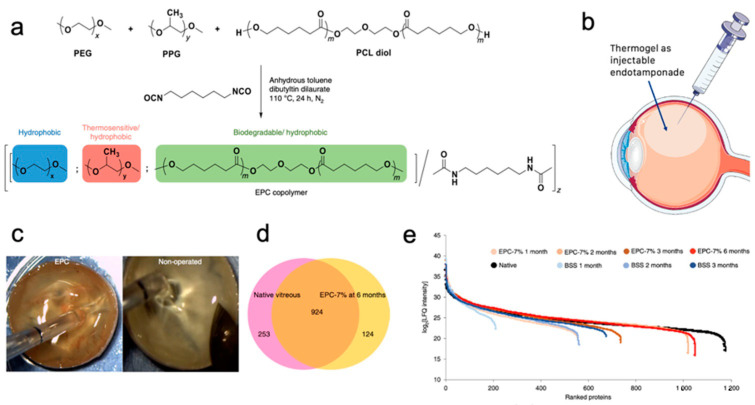
(**a**) Synthesis procedure of the EPC copolymer. (**b**) Schematic diagram illustrating thermogel use as endotamponade. (**c**) EPC vitreous-like body was formed that is similar to native vitreous. (**d**,**e**) Characterization of the vitreous-like body, showing (**d**) number of identified proteins which overlapped between native and EPC reformed vitreous and (**e**) ranked protein abundance across the native vitreous, reformed EPC, and BSS control proteome. Copyright © 2019 Springer *Nature* [106].

Vitreous humor fills the cavity in human eyes and is located between the lens and retina. It is mainly composed of water and considered a natural hydrogel in the eyes to support the retina. Aging could change the mechanical strength of vitreous humor and lead to the loss of supporting function, and vitreous humor is also removed during eye surgery to facilitate retinal detachment repair [104,105]. The challenge to utilize thermogels as a vitreous tamponade is to exert a tamponading force that can allow for retina re-adhesion and to ensure transparency within the vitreous space. In a new application, the PCL-based thermogels with low CGC developed by our group were applied to the treatment of retinal detachment (Figure 6) [106]. Biodegradable PEG/PPG/PCL multiblock thermogels (EPC) with 7 wt% of copolymer show long-term biocompatibility in rabbits and can effectively function as a tamponading agent in a non-human primate model of surgical detachment. Proteomics analysis indicates that majority of the top ten proteins in natural vitreous humor were present in EPC-7wt% thermogel, suggesting that the vitreous could be regenerated. EPC thermogel tested at a significantly higher polymer concentration of 12 wt% led to inflammation in the rabbit eye and increased intra-ocular pressure (IOP), which could be due to osmotic pressure. Another study used ultralow content of tetra-PEG hydrogel as vitreous tamponade which would eliminate the potential problem caused by osmotic pressure and swelling [107]. We studied the influence of EPC copolymer molecular weight and demonstrated the optimal molecular range for function as an endotamponade [108]. Furthermore, a PHA-based thermogel system was also developed and investigated, and shown to form a highly transparent vitreous substitute [109]. Our work shows that thermogels have great advantages and potential to be an ideal vitreous endotamponade in the future.

## 4. Conclusions and Perspectives

As a stimuli-responsive material, thermogels with a remarkable reverse phase transition feature at physiological temperatures have led to the emergence of several applications in the biomedical field. Numerous impressive works in recent years were cited and discussed in this review, ranging from a matrix for photothermal therapy to its use as a vitreous replacement. For the thermogelling mechanism, studies in different scales remind us that understanding thermogel properties is still a complex question that cannot be explained by any single mechanism at any single scale. Fortunately, as technology evolves, more new characterization and simulation techniques are available to help us understand these mechanisms more fully.

It has already been decades since the earliest batch of thermogel systems were developed. New thermogelling systems are still being discovered while currently existing thermogels are constantly being repurposed for new applications such as sprayable systems. The limitation of most thermogels today is that they can only respond to changes in temperature. The development of hydrogels that combine more sensing functions (e.g., hydrogels that combine pH-sensitivity, thermosensitivity, and photosensitivity with tunable parameters) and incorporate other bioactive moieties could allow for composite thermogel systems which are useful for more complex biological applications. Another inherent disadvantage of thermogels is their low mechanical strength due to the use of relatively weak physical crosslinking. The use of dual-network interpenetration may be able to impart higher mechanical strength to the thermogel, but it remains a challenge to form a highly biocompatible, high mechanical strength in situ gel while maintaining its original injectable properties. In the future, thermogels could have a greater impact in the growing field of tissue engineering. Novel techniques such as 3D printing can help to control the structure of thermogels at a more macroscopic level, enabling more accurate mimicry of tissue structure.

Although a number of experimental thermogels are developed, few have been commercialized so far. One reason could be the relatively high polymer concentration required for earlier generations of thermogels to gelate and release drug in a sustained manner, which could become an issue with frequent administration for chronic diseases. While there is a diversity of newer thermogels now, more effort is still needed to initiate and accelerate the long-term biocompatibility evaluation of thermogel polymers. The lessons from Oncogel™ tell us that we must be cautious when evaluating biomedical materials. Extensive biocompatibility and efficacy testing is still necessary for any thermogel that is actually moving into the clinical phase.

Thermogels are still amongst the most effective and useful in situ formed hydrogels due to the universality of tapping on physiological temperatures for biomedical applications. When the base thermogel material is combined with the latest new functionalities, we believe that it can add tremendous value to a range of biomedical applications. With the emergence of supramolecular hydrogels as promising materials, it will also be important to look beyond purely the depot forming property of thermogels and unearth other use cases that tap upon the dynamic and reversible nature of thermogelling polymers. 

## Figures and Tables

**Table 1:** Polymers with LCST that are commonly used in the biomedical field.

Polymer	Abbreviation	LCST (°C)	Reference
Poly(ethylene glycol)	PEG/PEO	106~115	[25]
Poly(propylene glycol)	PPG/PPO	10~40	[26]
Poly(vinylalcohol)	PVA/PVAl	125	[27]
Poly(N-isopropylacrylamide)	PNIPAM/PNIPAAM	32	[28]
Poly(methyl vinyl ether)	PMVE	28~34	[29,30]
Poly(N-vinyl caprolactam)	PNVCa/PVCL	30~50	[31,32]

**Table 2:** Thermogel systems for drug delivery.

Polymer Name	Drug	Application	Reference
Pluronic F-127	Brimonidine tartrate (BT), brinzolamide (BRZ), cyclosporine	Eye drop	[71]
PCGA-PEG-PCGA	Liraglutide	Treat type 2 diabetes	[73]
Elastin-like thermogel	Human C-peptide	Treat diabetic complications	[74]
PEG- PSMCU	lysozyme	In situ protein delivery	[75]
ND-based gelatin chitosan	VEGF	Growth factor bases therapies	[76]
PCL-based polyurethane thermogel	Anti-VEGF	Treat proliferative vascular diseases	[77]
PLGA-PEG-PLGA	ALA	Transdermal drug delivery	[78]
Alginate-g-PNIPAM	DOX	Treat cancer	[81]
PHB-based polyester	DOX, PTX	Treat Hepatocellular carcinoma	[82]
PDLLA-PEG-PDLLA	BP	Photothermal therapy	[84]

**Table 3:** Thermogel systems for tissue engineering.

Base Thermogel	Additional Functionalities	Polymer Effect	Application	Reference
Chitosan	GNPs	Electro-conductive	Cardiac repair	[94]
PNIPAM	PLGA, PVP/H_2_O_2_	Release oxygen	Cardiac repair	[95]
Poloxamer	Heparin	Release bFGF, NGF	Neurilemmal cells regeneration	[96]
Poloxamer	Heparin	Release GDNF	Spinal cord injury	[97]
P(NIPAAM-AA)	BMSCs	Scaffold for BMSCs	Osteochondral regeneration	[99]
PLGA-PEG-PLGA	BMSCs	Scaffold for BMSCs	Full-thickness cartilage repair	[100]
Chitosan- β-glycerophosphate	PCL	Scaffold for BMSCs	Bone tissue engineering	[101]
PCL-based thermogel	None	Vitreous tamponade	Retinal detachment	[106]
PHA-based thermogel	None	Vitreous tamponade	Retinal detachment	[109]
